# Biliary mucinous cystic neoplasm mimicking a hydatid cyst: a case report and literature review

**DOI:** 10.1186/s12876-019-1001-5

**Published:** 2019-06-24

**Authors:** Côme Tholomier, Yifan Wang, Olga Aleynikova, Tsafrir Vanounou, Jean-Sebastien Pelletier

**Affiliations:** 1 Division of General Surgery, Jewish General Hospital, 3755 Côte-Sainte-Catherine, Montréal, QC H3T 1E2 Canada; 2 Division of Urology, McGill University Health Center, Montréal, QC Canada; 3 Department of Pathology, Jewish General Hospital, Montreal, QC Canada

**Keywords:** Biliary cystadenoma, Biliary mucinous cystic neoplasm, Hydatid disease, Investigation, Management

## Abstract

**Background:**

Biliary mucinous cystic neoplasms are rare cystic lesions of the liver which carry pre-malignant potential. Given the scarcity of reports in the literature, they pose a considerable challenge to clinical management, particularly with regards to accurate pre-operative diagnosis.

**Case presentation:**

We present the case of a 37-year-old Tunisian woman who presented with subacute right upper quadrant pain and a large multi-loculated cystic lesion, most consistent with a hydatid cyst. She underwent an open right hepatectomy, and pathology surprisingly revealed a biliary mucinous cystadenoma. Herein, we review the current literature on biliary mucinous cystic neoplasms, with a particular emphasis on diagnostic investigations, key radiological features and optimal treatment modalities.

**Conclusion:**

Biliary mucinous cystic neoplasms require a high index of suspicion and should be managed with complete surgical resection, as conservative techniques are associated with high recurrence rates. Considering the potential for malignant transformation, periodical surveillance imaging is recommended in the post-operative period.

## Background

Biliary mucinous cystic neoplasms (BMCNs) are rare liver tumors that pose a considerable diagnostic challenge, as they can mimic various other liver lesions. Accurate pre-operative diagnosis may be difficult. A thorough understanding of the characteristic features of BMCNs and a high index of suspicion are essential to minimize the risk of malignant transformation or recurrence.

Herein, we present an interesting case of a 37-year-old female with a biliary mucinous cystic neoplasm mimicking a hydatid cyst. We review the current literature on BMCNs, including clinical presentation, diagnostic investigations (with an emphasis on radiological findings) and surgical management.

## Case presentation

**Fig. 1:**
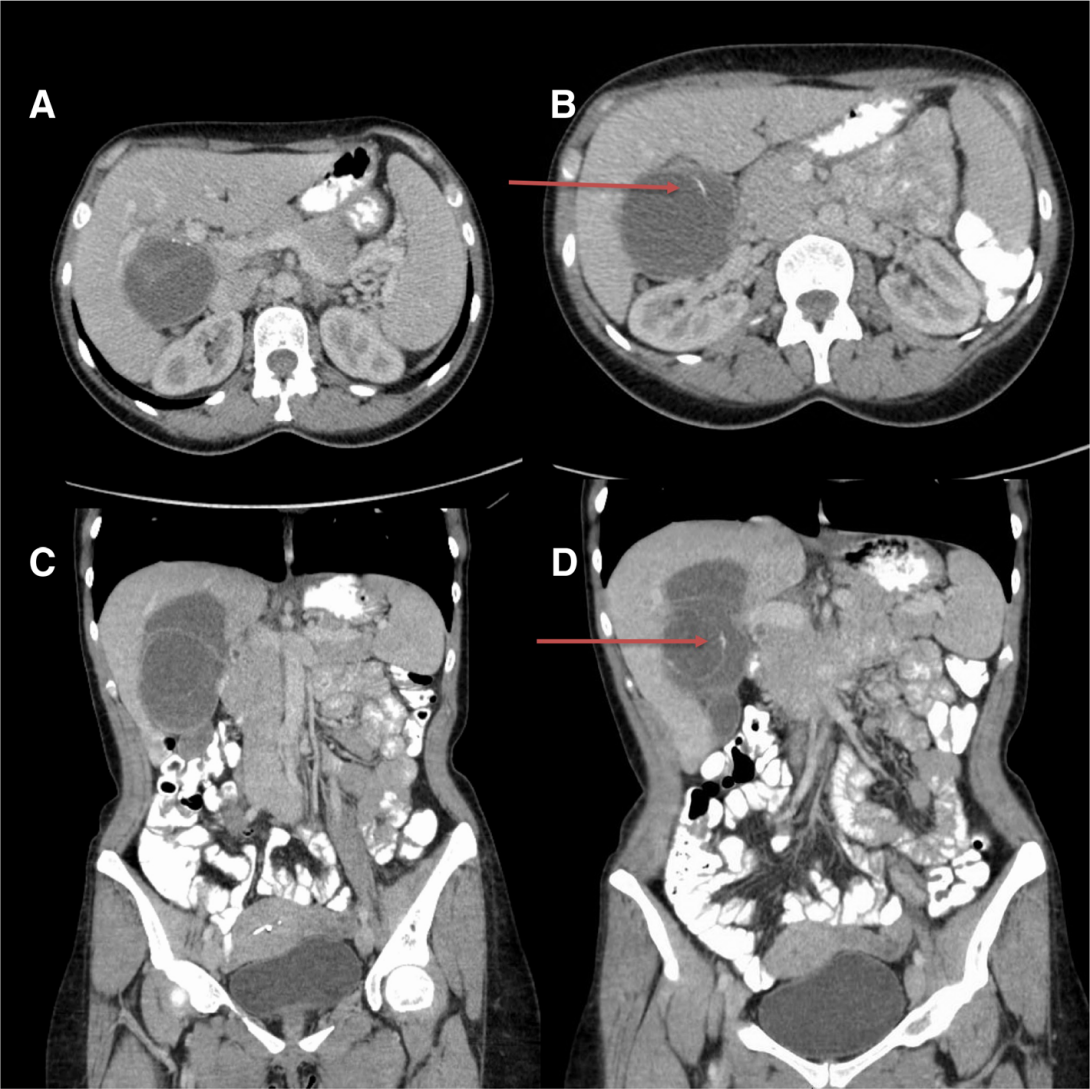
CT scan with oral and intravenous contrast in axial (1a and 1b) and coronal (1c and 1d) views, demonstrating a multi-loculated cystic mass embedded within segments V and VIII of the liver and causing deviation of the right portal vein. Arrows pointing towards the partially calcified septations

An otherwise healthy 37-year-old Tunisian woman presented to the emergency department with a 3–4 month history of right upper quadrant pain. She also reported general malaise and a 2–3 kg weight loss. She denied any jaundice, fever or infectious symptoms. She had been living in Tunisia up until the previous year, and had been exposed to dogs and sheep. On physical examination, she had mild right upper quadrant tenderness, without peritoneal signs. Computed tomography (CT) scan revealed a 7.1 × 6.5 × 10.7 cm complex, multi-loculated cystic mass with partially calcified septations, partially embedded within the liver (segments V/VIII) and causing deviation of the right portal vein (Fig. 1). There was no biliary dilatation or portal venous thrombosis. White blood cell count (5.5 × 10^9/L), eosinophil count (0.2 × 10^9/L) and liver function tests (Bilirubin 6, ALT 19, AST 19, ALP 48, GGT 15) were within normal limits. Tumor markers (including Ca19–9) and serum IgE level were not performed. She was discharged with outpatient hepatobiliary follow-up.

Given her history of proximity to livestock in an endemic area, the diagnosis of a hydatid cyst was strongly favored. However, echinococcosis and amoebiasis serologies were both negative. At the multidisciplinary tumor board discussion, the diagnostic value of a pre-operative biopsy was weighed against the risk of potential seeding. The Tropical Disease team deemed this lesion to be most likely a hydatid cyst, corresponding to a category CE 3A cyst according to the WHO classification [1]. Although only the minority (5–20%) of CE 3 cysts are sero-negative [2], the recommendation was to proceed with surgical resection, following a one-week pre-operative course of albendazole.

**Fig. 2:**
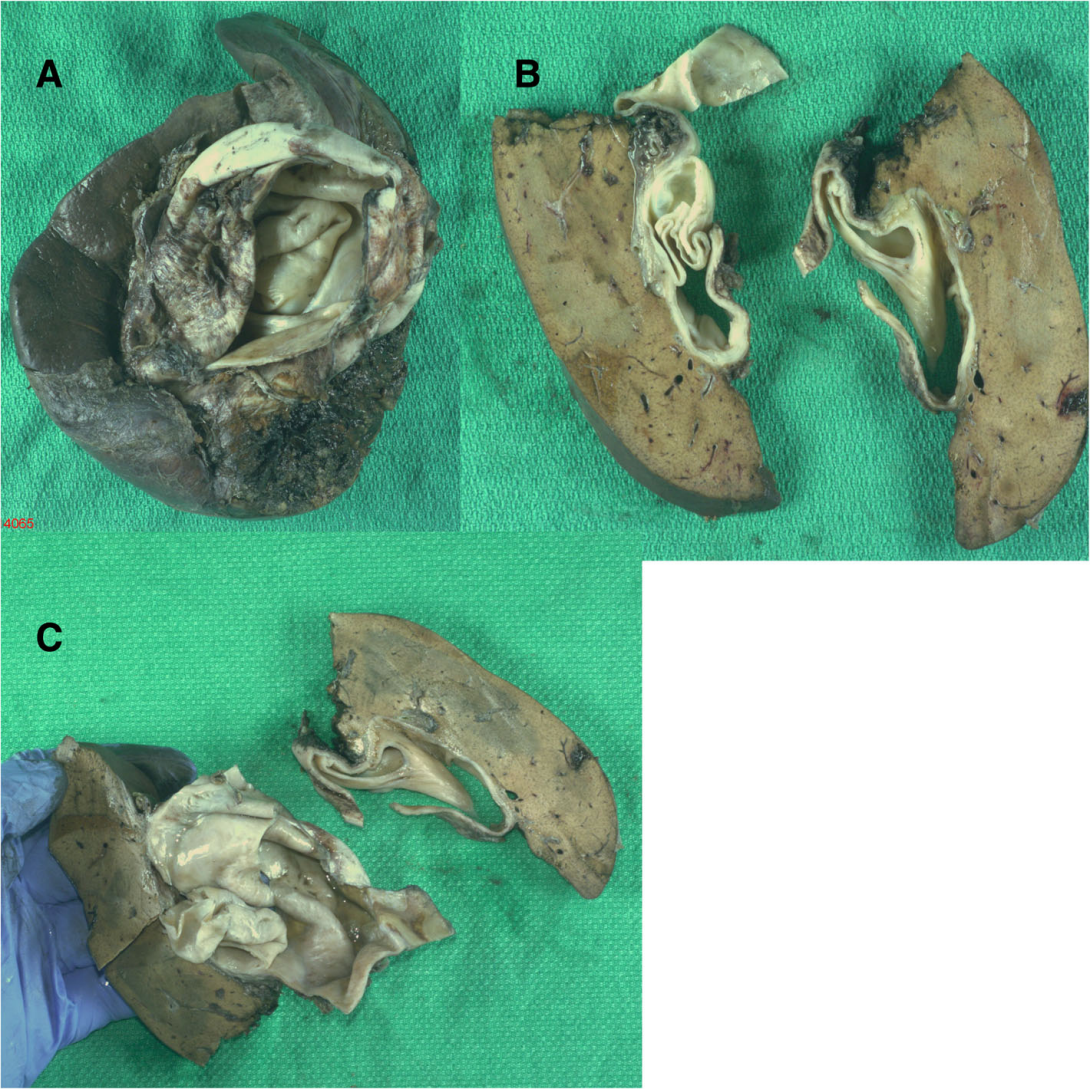
Gross morphology of the biliary mucinous cystic neoplasm (2a, 2b and 2c)

She underwent a right hepatectomy through a right subcostal incision considering the size of the lesion and its proximity to the right portal vein. Intra-operatively, the cyst appeared white, quite thickened, and intimately associated with the portal vein. It was opened on the back table and found to contain murky fluid as well as another cyst within, which was originally thought to represent a daughter cyst (Fig. 2). Her postoperative course was unremarkable, and she was discharged home on postoperative day 5. An abdominal ultrasound performed 6 months post-operatively showed no residual disease.

**Fig. 3:**
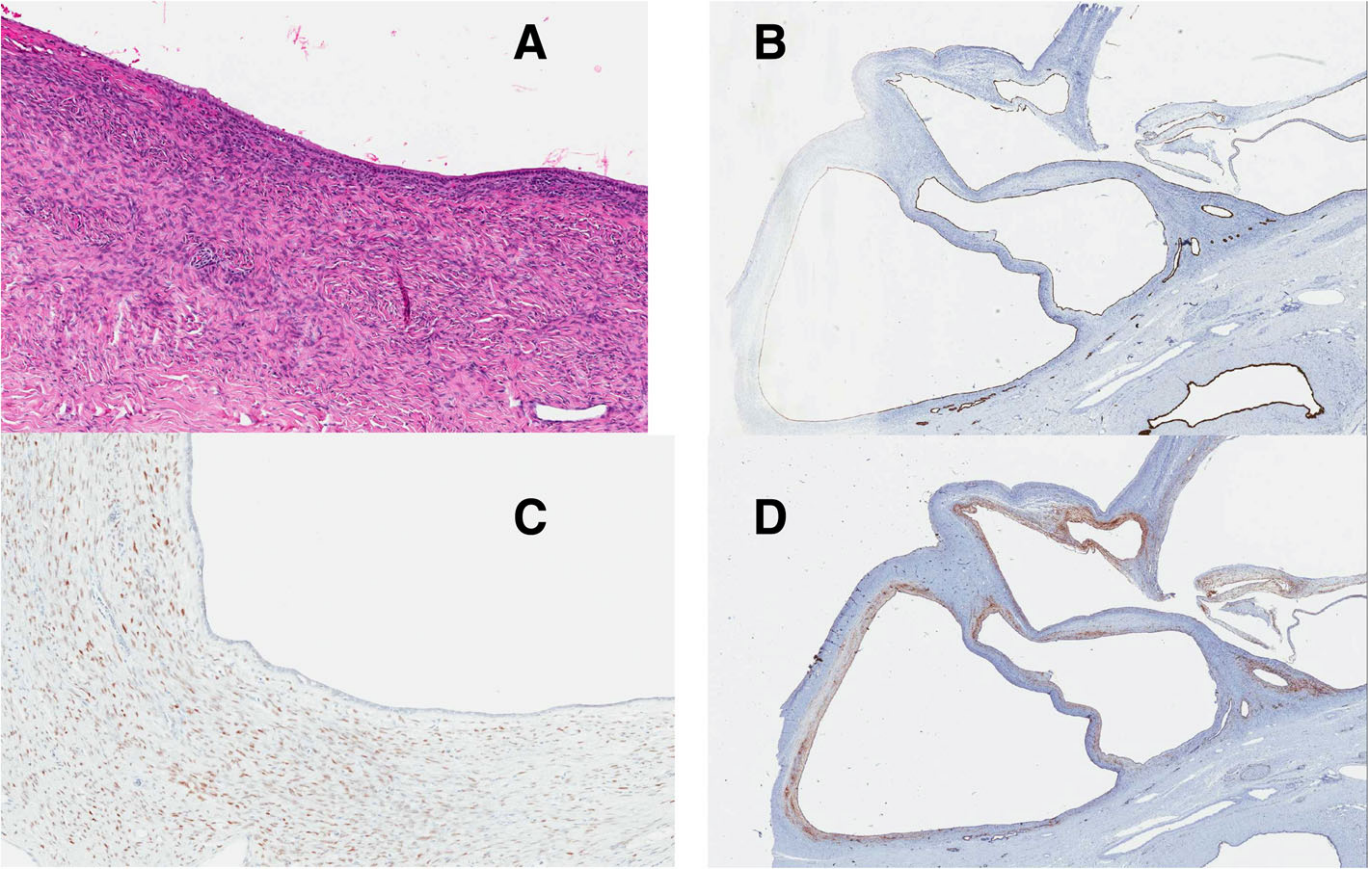
Light microscopy of the biliary mucinous cystic neoplasm showing the ovarian-type stroma with hematoxylin and eosin stain (3a). Immunohistochemical staining using Cytokeratin 19 (3b), estrogen receptor protein (3c) and CD10 (3d) highlighting the epithelium and ovarian stroma present in cystadenomas

Final pathology surprisingly revealed a biliary mucinous cystic neoplasm, which was completely excised with negative margins. It contained ovarian-type stroma and dystrophic calcifications. The cyst wall was composed of a single layer of cylindrical to flattened cuboidal epithelium (Fig. 3a). Cytokeratin 19, an epithelial marker, was positive (would be negative in hydatid cyst) (Fig. 3b). The estrogen receptor protein and CD10 were expressed, highlighting the ovarian-type stroma, which is typically seen in BMCN (Fig. 3c and d). Vimentin and PAX-8 were negative, thereby excluding gynecological origin. The remainder of the liver parenchyma and gallbladder were otherwise normal.

## Discussion and Conclusion

Biliary mucinous cystic neoplasms (BMCNs) are rare cystic lesions which predominantly occur in the liver, but can occasionally arise in the extra-hepatic biliary system. They account for less than 5% of non-parasitic liver cysts [3]. There are only a few hundred cases reported in the literature (Table 1). BMCNs occur almost exclusively (85–95%) in middle-aged females [7, 11]. Typically, patients are asymptomatic or present with an insidious onset of non-specific symptoms, which renders the clinical diagnosis, and distinction from hydatid cyst, challenging. However, a history of recent visit in an endemic area for hydatid disease can be an important clue. Acute onset of pain is usually secondary to intra-cystic hemorrhage or cyst rupture [10]. Contrary to hydatid cysts, BMCNs have been reported to increase in size during pregnancy and with oral contraceptives, suggesting hormonal dependency [5, 8].

**Table 1:** Review of contemporary published case series on biliary mucinous cystic neoplasms with more than 10 cases

	N	Sex	Age (years)	Size cm (mean)	Previous intervention	Operative Procedure and recurrences	Follow-up
Vogt et al. (2005) [4]	18	*F* = 18	48	12.5 (7–22)	10 patients (55%)	Enucleation [5]	Mean: 37 months
					Partial excision [6]	Sectionectomy [7] – *1 recurrence at 3 years*	*3/15 recurrences*
					Aspiration [8]	Hepatectomy [2]	
					Roux-en-Y [1]	Partial resection [7] – *2 recurrences at 13 months and 10 years*	
Thomas et al. (2005) [9]	18 BCA 1 BCAC	*F* = 18 *M* = 1	48	10.9 ± 4.4	8 patients (42%	Lobectomy [6]	Mean: 3.5 ± 4.2 years
					Aspiration or drainage [6]	Enucleation [10]	No mortality or recurrence
					Fenestration [11]	Bisegmentectomy [2]	
					Roux-en-Y [1]	Non-anatomic resection [1]	
						Fenestration + fulguration [1]	
Daniels et al. (2006) [12]	12	*F* = 12	50.5	12.7 (6.5–25)	5 patients (42%)	Enucleation + complete resection [8]	Unknown duration of follow-up
					Drainage [7]	Enucleation [7]	No mortality or recurrence
					Roux-en-Y [1]	Complete resection [1]	
Choi et al. (2010) [13]	17	*F* = 17	57	10.1 ± 6.6	Unknown	Hepatectomy [14]	N/A
						Unroofing or marsupialization [3]	
Erdogan et al. (2010) [15]	12 BCA 3 BCAC	*F* = 13 *M* = 2	45	8.4 ± 5.3 (3–19)	Unknown	Enucleation [6]	No early post-operative mortality
						Hepatectomy [10]	
Seo et al. (2010) [16]	13	*F* = 12 *M* = 1	55.3 ± 11.4	12.9 ± 6.3	None	Complete resection [17] – segmentectomies, lobectomies or enucleation	Median: 29 months
							No mortality or recurrence
Sang et al. (2011) [18]	19	*F* = 17 *M* = 2	44.2 ± 14.4	13.0 ± 8.1	5 patients (26%)	Hemihepatectomy [11] – *1 death from widespread metastasis at 8 months*	Median: 22.5 months
					Aspiration [10]	Sectionectomy [11]	*1/19 mortality*
					Fenestration [7]	Segmentectomy [11]	No recurrence
						Enucleation [3]	
						Fenestration [1]	
Martel et al. (2012) [19]	11 BCA 2 BCAC	*F* = 12 *M* = 1	51 ± 15.9	12.4 ± 4.6	None	Enucleation [10]	Median: 23.1 months
						Unroofing [7] – *1 death from MI 13 months post-op, 1 recurrence within 40 months*	*1/13 mortality*
							*1/13 recurrence*
						Liver resection [3]	
Pillai et al. (2012) [8]	13	*F* = 11 *M* = 2	46	(23–34)	7 (54%)	Hepatectomy [11]	Median: 22 months
					Partial cystoperi-	Segmentectomy [11]	No mortality or recurrence
					cystectomies [11]	Enucleation [3]	
					Marsupialisaton [1]		
					Fenestration [1]		
Ratti et al. (2012) [20]	12	*F* = 12	45 ± 9.4	N/A	6/12 (50%) ➔ Unroofing followed by complete resection after post-op diagnosis (median delay of 31 days)	Hepatectomy [6]	Median: 16 months
						Lobectomy [3]	No mortality or recurrence
Wang et al. (2012) [21]	20	*F* = 19 *M* = 1	44.2 ± 13.1	13.0 (4.4–32)	Unknown	Liver resection [22]	N/A
Chen et al. (2014) [23]	35 BCA 4 BCAC	*F* = 30 *M* = 9	53.7 ± 14.5	N/A	None	Enucleation [24]	Median: 20.5 months
						Hepatectomy [25]	*1/4 recurrence in a patient with BCAC*
							No mortality or recurrence for BCA
Wang et al. (2014) [26]	13 BCA 1 BCAC	*F* = 11 *M* = 3	48	10.4 ± 6.3	None	Lobectomy [8]	Median: 54 months
							*1/1 recurrence for BCAC 2 years post-op*
						Hepatectomy [2]	No mortality or recurrence for BCA
						Partial hepatic resection [7]	
						Non-anatomic resection [1]	
Albores-Saavedra et al. (2015) [27]	18 BCA 2 BCAC	*F* = 18 *M* = 2	38.2	11 (4–29)	None	Enucleation [4]	Range 2–11 years
						Lobectomy [10]	*3/15 recurrences for BCA patients*
						Whipple resection [2]	
						Local excision [2]	
Lee et al. (2015) [28]	19	*F* = 16 *M* = 3	57	8.5 (2.5–21)	None	Complete resection: anatomic or enucleation [29]	Median: 51.3 months
						Partial resection: fenestration, marsupialization, partial excision [5] – *5 (62.5%) recurrences, 1 death 32 days postop*	Median DFS: 3.7 months (for partial resection)
Arnaoutakis et al. (2015) [30]	221	*F* = 199 *M* = 22	51.2 ± 15.1	Median: 10.0 IQR: 7–13	Cyst aspiration (19.8%) Sclerosis (2%)	Partial hepatectomy (116, 52.5%)	10-year follow-up: 84% patients alive with BCA 31/221 (16.7%)
						Hepatectomy (70, 31.7%) – *15/186 (8.1%) recurrences*	
	Total: 248 BCAC: 27					Unroofing/partial excision (34, 15.4%) – *16 (45.6%) recurrences*	recurrences 50 (22.6%) early post-operative complications
						Liver transplant (1, 0.4%)	

*BCA* Biliary cystadenoma (biliary mucinous cystic neoplasia), *BCAC* Biliary cystadenocarcinoma (biliary mucinous cystic neoplasia with invasive carcinoma), *DFS* Disease-free survival, *OS* Overall survival, *RFS* Relapse-free survival, *F* Female, *M* Male, *MI* Myocardial Infarction, *IQR* Inter-quartile range

Full articles are available via PubMed and originally in English

When possible, biliary cystadenocarcinoma cases were removed from analysis given focus of this manuscript on biliary cystadenoma

Biliary mucinous cystic neoplasms are histologically divided into two types, depending on the presence of mesenchymal stroma, which is a subepithelial stroma resembling ovarian stroma [11]. BMCNs with mesenchymal stroma are considered to carry a favorable prognosis, and are exclusively seen in women [5]. Those without mesenchymal stroma are more susceptible to malignant transformation and are associated with a poorer prognosis. The cyst fluid may be mucinous or serous. Blood-tinged cyst fluid raises concern for a malignant component [6]. Cyst sizes are largely variable, with reports ranging from 1.5 to 35 cm [4, 29]. The differential diagnosis of BMCN includes hydatid cysts, simple liver cysts, abscesses, hematomas and mucinous cystic neoplasms with associated invasive carcinoma (cystadenocarcinomas) [3]. Differentiating BMCN from these other entities is critical, in light of the high recurrence rate if incompletely excised, and potential for malignant transformation.

### Radiological findings

Ultrasonography (US), CT and magnetic resonance imaging (MRI) are the most commonly used imaging modalities in the workup of BMCN [31]. They typically appears as a multi-loculated cyst with a well-defined, thick capsule and internal septations. Solid papillary projections, internal septae and wall enhancement upon contrast administration are other characteristic features [17]. While hydatid cysts preferentially affect the right lobe, a lesion within the left liver is more suggestive of BMCN [14, 17, 29]. Upstream dilatation of adjacent intra-hepatic biliary radicles can occur secondary to mass effect. Demonstrable communication of the cyst with the biliary system is a specific, albeit rarely observed, finding [32]. Therefore, pre-operative ERCP (endoscopic retrograde cholangiopancreatography) and PTC (percutaneous transhepatic cholangiography or cystography) can potentially help in the diagnosis of cystadenoma if a communication is seen. It is especially important if a patient presents with jaundice since it can reveal biliary compression by the tumor [33]. Finally, intra-operative cholangiography (IOC), while also allowing fluid sampling for cytology, may be useful to diagnose extrahepatic biliary tree cystadenoma [25]. Nevertheless, most BMCNs will have no identifiable biliary connection, even intra-operatively [6].

The presence of an irregular and thickened cystic wall, hypervascular mural solid nodules, thick calcifications and papillary projections may suggest an increased risk of malignancy [34]. However, imaging findings cannot reliably differentiate cystadenoma from cystadenocarcinoma. Despite certain radiological findings which may favor the diagnosis of BMCN, the sensitivity of pre-operative diagnosis remains low (30%). As such, a high index of suspicion should be maintained when imaging findings are non-diagnostic.

The radiological appearance of hepatic hydatid disease is also quite variable. On US, the cyst wall is usually seen as double echogenic lines separated by a hypo-echogenic layer. Calcifications can be present in the cyst wall, although internal calcifications are also seen. Hydatid sand may be visualized as small echogenic foci falling to the most dependent portion of the cyst (i.e. snowstorm sign) [22, 35]. Daughter cysts are another feature of hydatid disease. They appear as cysts within a cyst and are usually separated by multiple septa in a honeycomb pattern [22]. Occasionally, membranes of broken daughter vesicles appear as “serpentine linear structures” inside the hydatid matrix; this feature is highly specific for hydatid disease [36]. CT findings are overall similar to the ones observed with US. On T2 MRI, hydatid cysts may have a low signal intensity rim, which has been proposed as a characteristic feature of this disease [36].

### Adjunct investigations

Core needle biopsy is not routinely recommended, because of its low diagnostic accuracy and the risk of seeding and dissemination, in case of malignancy [24]. In addition, biopsy should be avoided when the diagnosis of a hydatid cyst remains a possibility, as hydatid cyst rupture is associated with significant risk of anaphylaxis [37]. While elevated CEA and Ca-19-9 levels in the serum or cyst fluid may be helpful, a normal level does not exclude BMCN [38]. In fact, some case series report no significant difference between these levels when compared to simple hepatic cysts [13]. Serological tests may be useful in the workup of a suspected echinococcal cyst, although diagnosis may prove challenging in the face of a weak immune response [39].

### Management

Management of biliary mucinous cystic neoplasms is dictated by two key concerns. First, if misdiagnosed as a simple cyst or a hydatid cyst, incomplete excision (such as deroofing, marsupialization or partial resection) may be undertaken. Incomplete excision of BMCN is associated with a high recurrence rate, with some authors reporting recurrence rates over 90% [6, 40]. Thus, complete excision is indicated, and recurrence of a hepatic cyst following partial resection should raise suspicion for a BMCN [9]. In the case of hepatic hydatid disease, surgical management is recommended to avoid complications such as exophytic growth leading to pressure or mass effects on nearby structures (such as bile ducts, portal or hepatic veins), cyst rupture leading to peritonitis, bacterial infection which can result in liver abscesses, transdiaphragmatic thoracic involvement as well as hematogenous dissemination [14]. A pre-operative course of anthelmintics (e.g. albendazole) is recommended to achieve sterilization and to reduce the risk of recurrence post-operatively [41, 42].

Second, because differentiating BMCN from cystadenocarcinoma pre-operatively is exceedingly difficult, complete surgical resection remains the gold standard [38, 43]. The risk of malignant transformation to cystadenocarcinoma supports the role of surgical resection. Lewis et al. published a series of 15 BMCNs for which formal resection was undertaken, and reported few complications and no recurrences [44]. Table 1 summarizes the major characteristics of the largest contemporary case series on BMCNs. It emphasizes that complete resection is a safe and efficacious method to treat cystadenoma with good outcomes and low risk of recurrence. When the diagnosis is unclear pre-operatively between hydatid cyst and BMCN, we would recommend complete resection, as it would treat both diseases, and minimize the risk of recurrence.

Enucleation is a viable alternative in cases in which formal resection would be technically difficult or associated with excessive morbidity [45, 46]. Although only small case series are available, some have shown comparable results compared to formal hepatectomy, with few recurrences [9, 47]. However, given the large size of these lesions and the resulting distorted anatomy, enucleation remains technically challenging and must be approached with caution.

Finally, given the high rate of recurrence and the potential risk for malignant transformation [48], surveillance imaging with ultrasound or CT scan at regular intervals is recommended post-operatively [20, 49].

## Discussion

Biliary mucinous cystic neoplasms are rare liver neoplasms, which may strongly mimic a hydatid cyst in patients with a contributory history. Pre-operative differentiation on the basis of radiological, biochemical and serological features is often unreliable. Suspicious multilocular cystic lesions should be managed with complete surgical resection, as conservative techniques are associated with high recurrence rates in cases of BMCN. Considering the potential for malignant transformation, periodical surveillance imaging is recommended in the post-operative period.

## Data Availability

All data generated or analyzed during this study are included in this published article.

## Abbreviations

CEA: Carcinoembryonic antigen
CT: Computed Tomography
ERCP: Endoscopic retrograde cholangiopancreatography
IOC: Intra-operative cholangiography
MRI: Magnetic resonance imaging
PTC: Percutaneous transhepatic cholangiography or cystography
US: Ultrasonography
WHO: World Health Organization

## References

[1] Group WHOIW (2003). International classification of ultrasound images in cystic echinococcosis for application in clinical and field epidemiological settings. Acta Trop.

[2] Rinaldi F, Brunetti E, Neumayr A, Maestri M, Goblirsch S, Tamarozzi F (2014). Cystic echinococcosis of the liver: a primer for hepatologists. World J Hepatol.

[3] Walt AJ (1977). Cysts and benign tumors of the liver. Surg Clin North Am.

[4] Vogt DP, Henderson JM, Chmielewski E (2005). Cystadenoma and cystadenocarcinoma of the liver: a single center experience. J Am Coll Surg.

[5] Wheeler DA, Edmondson HA (1985). Cystadenoma with mesenchymal stroma (CMS) in the liver and bile ducts. A clinicopathologic study of 17 cases, 4 with malignant change. Cancer..

[6] Florman SS, Slakey DP (2001). Giant biliary cystadenoma: case report and literature review. Am Surg.

[7] Emre A, Serin KR, Ozden I, Tekant Y, Bilge O, Alper A (2011). Intrahepatic biliary cystic neoplasms: surgical results of 9 patients and literature review. World J Gastroenterol.

[8] Ahanatha Pillai S, Velayutham V, Perumal S, Ulagendra Perumal S, Lakshmanan A, Ramaswami S (2012). Biliary cystadenomas: a case for complete resection. HPB Surg.

[9] Thomas KT, Welch D, Trueblood A, Sulur P, Wise P, Gorden DL (2005). Effective treatment of biliary cystadenoma. Ann Surg.

[10] Sun Y, Lu X, Xu Y, Mao Y, Yang Z, Sang X (2010). Spontaneous rupture of a giant hepatobiliary serous cystadenoma: report of a case and literature review. Hepatol Int.

[11] Davies W, Weiland L, Batts K, Nagorney DM (1999). Intrahepatic biliary cystadenomas with and without mesenchymal stroma. HPB.

[12] Daniels JA, Coad JE, Payne WD, Kosari K, Sielaff TD (2006). Biliary cystadenomas: hormone receptor expression and clinical management. Dig Dis Sci.

[13] Choi HK, Lee JK, Lee KH, Lee KT, Rhee JC, Kim KH (2010). Differential diagnosis for intrahepatic biliary cystadenoma and hepatic simple cyst: significance of cystic fluid analysis and radiologic findings. J Clin Gastroenterol.

[14] Pedrosa I, Saiz A, Arrazola J, Ferreiros J, Pedrosa CS (2000). Hydatid disease: radiologic and pathologic features and complications. Radiographics..

[15] Erdogan D, Kloek J, Lamers WH, Offerhaus GJ, Busch OR, Gouma DJ (2010). Mucinous cystadenomas in liver: management and origin. Dig Surg.

[16] Seo JK, Kim SH, Lee SH, Park JK, Woo SM, Jeong JB (2010). Appropriate diagnosis of biliary cystic tumors: comparison with atypical hepatic simple cysts. Eur J Gastroenterol Hepatol.

[17] Kim JY, Kim SH, Eun HW, Lee MW, Lee JY, Han JK (2010). Differentiation between biliary cystic neoplasms and simple cysts of the liver: accuracy of CT. AJR Am J Roentgenol.

[18] Sang X, Sun Y, Mao Y, Yang Z, Lu X, Yang H (2011). Hepatobiliary cystadenomas and cystadenocarcinomas: a report of 33 cases. Liver Int.

[19] Martel G, Alsharif J, Aubin JM, Marginean C, Mimeault R, Fairfull-Smith RJ (2013). The management of hepatobiliary cystadenomas: lessons learned. HPB.

[20] Ratti F, Ferla F, Paganelli M, Cipriani F, Aldrighetti L, Ferla G (2012). Biliary cystadenoma: short- and long-term outcome after radical hepatic resection. Updat Surg.

[21] Wang C, Miao R, Liu H, Du X, Liu L, Lu X (2012). Intrahepatic biliary cystadenoma and cystadenocarcinoma: an experience of 30 cases. Dig Liver Dis.

[22] Gharbi HA, Hassine W, Brauner MW, Dupuch K (1981). Ultrasound examination of the hydatic liver. Radiology..

[23] Chen YW, Li CH, Liu Z, Dong JH, Zhang WZ, Jiang K (2014). Surgical management of biliary cystadenoma and cystadenocarcinoma of the liver. Genet Mol Res.

[24] Simo KA, McKillop IH, Ahrens WA, Martinie JB, Iannitti DA, Sindram D (2012). Invasive biliary mucinous cystic neoplasm: a review. HPB.

[25] Hanazaki K (1996). Intrahepatic biliary cystadenoma demonstrated by intraoperative cholangiography. Hepatogastroenterology..

[26] Wang K, Kong F, Dong M, Zhou J, Li Y (2014). Diagnosis and treatment of intrahepatic biliary cystadenoma: experience with 14 cases in a single center. Med Oncol.

[27] Albores-Saavedra J, Cordova-Ramon JC, Chable-Montero F, Dorantes-Heredia R, Henson DE (2015). Cystadenomas of the liver and extrahepatic bile ducts: morphologic and immunohistochemical characterization of the biliary and intestinal variants. Ann Diagn Pathol.

[28] Lee CW, Tsai HI, Lin YS, Wu TH, Yu MC, Chen MF (2015). Intrahepatic biliary mucinous cystic neoplasms: clinicoradiological characteristics and surgical results. BMC Gastroenterol.

[29] Soares KC, Arnaoutakis DJ, Kamel I, Anders R, Adams RB, Bauer TW (2014). Cystic neoplasms of the liver: biliary cystadenoma and cystadenocarcinoma. J Am Coll Surg.

[30] Arnaoutakis DJ, Kim Y, Pulitano C, Zaydfudim V, Squires MH, Kooby D (2015). Management of biliary cystic tumors: a multi-institutional analysis of a rare liver tumor. Ann Surg.

[31] Mortele KJ, Ros PR (2001). Cystic focal liver lesions in the adult: differential CT and MR imaging features. Radiographics..

[32] Borhani AA, Wiant A, Heller MT (2014). Cystic hepatic lesions: a review and an algorithmic approach. AJR Am J Roentgenol.

[33] Manouras A, Markogiannakis H, Lagoudianakis E, Katergiannakis V (2006). Biliary cystadenoma with mesenchymal stroma: report of a case and review of the literature. World J Gastroenterol.

[34] Marrone G, Maggiore G, Carollo V, Sonzogni A, Luca A (2011). Biliary cystadenoma with bile duct communication depicted on liver-specific contrast agent-enhanced MRI in a child. Pediatr Radiol.

[35] Lewall DB, McCorkell SJ (1985). Hepatic echinococcal cysts: sonographic appearance and classification. Radiology..

[36] von Sinner WN (1991). New diagnostic signs in hydatid disease; radiography, ultrasound, CT and MRI correlated to pathology. Eur J Radiol.

[37] Grant A, Neuberger J (1999). Guidelines on the use of liver biopsy in clinical practice. British Society of Gastroenterology. Gut.

[38] Koffron A, Rao S, Ferrario M, Abecassis M (2004). Intrahepatic biliary cystadenoma: role of cyst fluid analysis and surgical management in the laparoscopic era. Surgery..

[39] Pakala T, Molina M, Wu GY (2016). Hepatic Echinococcal cysts: a review. J Clin Transl Hepatol.

[40] Short WF, Nedwich A, Levy HA, Howard JM (1971). Biliary cystadenoma. Report of a case and review of the literature. Arch Surg.

[41] Malik AA, Bari SU, Amin R, Jan M (2010). Surgical management of complicated hydatid cysts of the liver. World J Gastrointest Surg.

[42] Dehkordi AB, Sanei B, Yousefi M, Sharafi SM, Safarnezhad F, Jafari R, et al. Albendazole and treatment of hydatid cyst, review of literature. Infect Disord Drug Targets. 2018;19(2):101–4. 10.2174/1871526518666180629134511 29956639

[43] Lauffer JM, Baer HU, Maurer CA, Stoupis C, Zimmerman A, Buchler MW (1998). Biliary cystadenocarcinoma of the liver: the need for complete resection. Eur J Cancer.

[44] Lewis WD, Jenkins RL, Rossi RL, Munson L, ReMine SG, Cady B (1988). Surgical treatment of biliary cystadenoma. A report of 15 cases. Arch Surg.

[45] Morris M, Anderson C, Drake L, Redfield S, Subramony C, Vanderlan W (2012). Giant biliary cystadenoma. J Surg Case Rep.

[46] Dixon E, Sutherland FR, Mitchell P, McKinnon G, Nayak V (2001). Cystadenomas of the liver: a spectrum of disease. Can J Surg.

[47] Treska V, Ferda J, Daum O, Liska V, Skalicky T, Bruha J (2016). Intrahepatic biliary cystadenoma-diagnosis and treatment options. Turk J Gastroenterol.

[48] Woods GL (1981). Biliary cystadenocarcinoma: case report of hepatic malignancy originating in benign cystadenoma. Cancer..

[49] Ferraguti DA, McGetrick M, Zendejas I, Hernandez-Gonzalo D, Cystadenoma G-PRM (2017). A rare hepatic tumor in a child. Front Pediatr.

